# Pursuit and Evasion Strategy of a Differential Game Based on Deep Reinforcement Learning

**DOI:** 10.3389/fbioe.2022.827408

**Published:** 2022-03-22

**Authors:** Can Xu, Yin Zhang, Weigang Wang, Ligang Dong

**Affiliations:** ^1^ School of Statistics and Mathematics, Zhejiang Gongshang University, Hangzhou, China; ^2^ School of Information and Electronic Engineering, Sussex Artificial Intelligence Institute, Zhejiang Gongshang University, Hangzhou, China; ^3^ Collaborative Innovation Center of Statistical Data Engineering, Technology and Application, Zhejiang Gongshang University, Hangzhou, China

**Keywords:** dog sheep game, deep reinforcement learning, deep Q network, deep deterministic policy gradient, differential game

## Abstract

Since the emergence of deep neural network (DNN), it has achieved excellent performance in various research areas. As the combination of DNN and reinforcement learning, deep reinforcement learning (DRL) becomes a new paradigm for solving differential game problems. In this study, we build up a reinforcement learning environment and apply relevant DRL methods to a specific bio-inspired differential game problem: the dog sheep game. The dog sheep game environment is set on a circle where the dog chases down the sheep attempting to escape. According to some presuppositions, we are able to acquire the kinematic pursuit and evasion strategy. Next, this study implements the value-based deep Q network (DQN) model and the deep deterministic policy gradient (DDPG) model to the dog sheep game, attempting to endow the sheep the ability to escape successfully. To enhance the performance of the DQN model, this study brought up the reward mechanism with a time-out strategy and the game environment with an attenuation mechanism of the steering angle of sheep. These modifications effectively increase the probability of escape for the sheep. Furthermore, the DDPG model is adopted due to its continuous action space. Results show the modifications of the DQN model effectively increase the escape probabilities to the same level as the DDPG model. When it comes to the learning ability under various environment difficulties, the refined DQN and the DDPG models have bigger performance enhancement over the naive evasion model in harsh environments than in loose environments.

## 1 Introduction

Bio-inspired differential games have received much attention in recent years due to their broad applications in surveillance (1975) (Lewin and Breakwell, 1975) and air combat (1978, 1981) (Shinar and Gutman, 1978; Shinar, 1981). Differential games were initially proposed by Isaacs (1965). It is a game theory which solves dynamic game problems through differential equations. In each case, autonomous agents divided into two sides, pursuers and evaders, are against each other. This article focuses on the dog sheep game, which is a bio-inspired differential pursuit–evasion game in a constrained environment. The dog chases down the escaping sheep on the circle, while the sheep manages to run away without getting caught. According to the critical game conditions, we analyze the kinematics of pursuit and evasion strategy. The result shows the circle can be divided into three parts, each representing the position set of sheep based on whether the sheep is able to escape.

Designing reasonable differential equations from a given game scenario is the theme of differential games. Recently, deep reinforcement learning (DRL) methods have applied DNNs to learn agent strategies, which guide agents to interact with the environment. Earlier DRL methods like the deep Q network (DQN) model developed by DeepMind (2013, 2015) (Mnih et al., 2013; Mnih et al., 2015) which combines deep learning with Q-Learning (1992) (Watkins and Dayan, 1992) not only surpassed human-level performance but also outperformed many reinforcement learning methods. The deep deterministic policy gradient (DDPG) model (2015) (Lillicrap et al., 2015) uses off-policy data and the Bellman equation to learn the Q value, and uses the Q-function to learn the policy. The benefit of DRL methods is that it avoids the chaos and potential confusion of manually designed differential equations of each game scenario. Once the game scenario is set up, deep reinforcement learning methods allow us to directly acquire the kinematic strategy of agents end to end. This allows us to directly obtain the pursuit and evasion policy in a much easier way.

This study explores the use of DRL methods for guiding ignorant sheep to escape from the circle, rather than a handcraft for each game scenario. Powerful as the DQN model may seem, adjustments and refinements are still required when implementing it in various game scenarios such as the experience replay and the carefully designed reward mechanism. In this study, a delicate reward mechanism is applied to help train the DQN model. According to the cost function in the differential pursuit–evasion game (Yong, 2014), a time-out reward strategy is introduced to help agents obtain the optimal policy that minimizes the cost function. Due to its discrete action space, it is not practical to let the sheep choose the exact steering angle in the dog sheep game with limited computing power. Therefore, an attenuation mechanism of the steering angle is proposed based on the kinematic theory of the dog sheep game. This reduces the action space dimension significantly without compromising the chance of evasion for the sheep too much. Another way to overcome the defect of the discrete action space is to adopt different methods. This study adopts the powerful deep deterministic policy gradient (DDPG) model, which is actor-critic based and model-free. When simulating the dog sheep game, the model is trained with no knowledge in advance. Generally speaking, we mainly focus on two DRL methods to this end, DQN and DDPG, each with a different rationality. Later, we make some adjustments and refinements to improve the performance of DRL methods.

This work addresses the aforementioned research problems by implementing DRL methods for pursuit–evasion problems. Considering the dog sheep game is set in a constrained environment, the first problem this study addresses is solving the kinematic pursuit and evasion policies based on the differential pursuit–evasion game theory. DRL has shown its powerful performance in games, and the question is whether it is possible to endow the sheep the ability to escape. Therefore, the second problem is to set up a reinforcement learning environment and implement appropriate DRL methods to this particular differential game. However, some DRL methods may need adjustments when being applied to various scenarios. Hence, the third problem is to optimize the performance of DRL methods with the idea from the differential games theory. We summarize the contributions of this work as follows:

• For the first problem, by finding the equilibrium point in the dog sheep game, this study successfully establishes the kinematic pursuit and evasion policies.
• For the second problem, a delicate reward mechanism is introduced according to related theories of differential pursuit–evasion games.
• For the third problem, due to the defect of DQN whose the action space is discrete, an attenuation mechanism of the steering angle is proposed.
• By quantifying the environment difficulty, this study evaluates the performance and learning ability of our refined DQN model and DDPG model and other baseline models.

The rest of the article is organized as follows. In Section 3, the mathematical form of differential pursuit–evasion game theory is introduced. The kinematic pursuit and evasion strategy is solved in Section 4, while the implementation of DRL methods for evasion policy learning is elaborated in Section 5.

## 2 Related Work

Researchers have studied the implementations of DRL methods for differential pursuit–evasion games and improved them in many aspects. Isaacs (1965) first proposed the theory of differential games. In his book, Isaacs proposed a classic “homicidal chauffeur” problem which is a classic differential pursuit–evasion game. In this game, a slow but maneuverable pedestrian is against a driver with a faster but less maneuverable vehicle, attempting to run over the pedestrian. Merz (1971) presented the complete solution for the “homicidal chauffeur” game. Another classic game scenario is the game in constrained environments. For example, agents in Sundaram et al.’s (2017) study are constrained to road networks. The control policies of our research are based on the kinematic pursuit and evasion game theory. Apart from differential games, kinematic analysis is applied to various research areas, such as robotic control (2021) (Liu et al., 2021a; Liu et al., 2021b; Xiao et al., 2021; Zhao et al., 2022).

Deep neural networks have shown their advantages over traditional methods in multiple research areas. For example, they have been adopted in semantic analysis (2021) (Chen et al., 2021a; Chen et al., 2021b; Jiang et al., 2021b; Chen et al., 2021c) and image recognitions (2021) (Hao et al., 2021; Jiang et al., 2021a; Yang et al., 2021). The DRL method, which combines DNNs and reinforcement learning, was first proposed by DeepMind (2013, 2015) (Mnih et al., 2013; Mnih et al., 2015). Their method called DQN is the combination of Q-learning and deep neural network. It shows excellent performance in the Atari game. The emergence of the DQN leads to a number of similar research studies based on DRL methods. Shao et al. (2019) surveyed the application of DRL methods in video games. Value-based, policy gradient, and model-based DRL methods are applied to various video games such as Atari, Minecraft, and StarCraft. For example, Wei et al. (2018) employed convolutional neural networks trained with refined DQN to play the snake game. DRL has been a long-standing research area when it comes to artificial intelligence in differential games. Jiang et al. (2020) introduced an approximate soft policy iteration-based reinforcement learning method which used a value neural network to provide cooperative policy for two pursuers versus an evader. Lin (1992) proved that the experience replay helps the network train faster and smoother.

To overcome the defect of the discrete action space, researchers hypothesized several methods with a continuous action space. The actor-critic (A3C) method is utilized in many related research studies. For example, Perot et al. (2017) adopted the A3C with CNN + LSTM as the state encoder to play racing games. Lixin Wang et al. (2019) used a fuzzy deterministic policy gradient algorithm to obtain the specific physical meaning for policy learning in a pursuit–evasion game. Lillicrap et al. (2015) first introduced the DDPG methods with the continuous action space. Maolin Wang et al. (2019) implemented the DDPG model to an open pursuit–evasion environment to learn the control strategy. Several researchers (Lowe et al., 2020; Singh et al., 2020; Wan et al., 2021) proposed an actor-critic multi-agent DDPG algorithm to preprocess actions of multiple agents in the virtual environment.

## 3 Preliminary

This article focuses on the dog sheep game, in which the dog is the pursuer and the sheep is the evader. The game takes place in a circle. The dog is only allowed to pursue inside the circle, while the sheep is randomly born inside the circle. The distance between the sheep and the center of the circle is non-decreasing; otherwise, it would cause confusion when solving the kinematic evasion strategy. The objective of the sheep is to obtain an evasion strategy that maximizes its chance of escaping, while the objective of the dog is exactly the opposite.

The dog sheep game perfectly fits the differential game theory (1992) (Lin, 1992). In a differential pursuit–evasion game, the pursuer and the escaper have their own strategy and target. The following x,X() each refers to the state and the state trajectory; $\alpha_{1},A_{1},u_{2},U_{2}$ each represents the strategy used and the strategy set for the pursuer and the escaper; and t and $𝓣\left(x,\alpha_{1}\right)$ are the game time and the capturing time of a game, respectively.

$$\begin{array}{c} \{\begin{array}{cc} \dot{X}\left(t\right)=f\left(X\left(t\right),\alpha_{1}\left[u_{2}\right]\left(t\right),u_{2}\left(t\right)\right) & t\in \left[0,\infty \right)\\ X\left(0\right)=x & x\in R^{n} \end{array} \end{array}.$$ (1)

For the pursuer, let M be a moving target set. For any $\left(t,x\right)\in R^{+}\times R^{n}$ , the objective is to find an $\alpha_{1}\in A_{1}$ such that for any $u_{2}\in U_{2}$ ,

$$\begin{array}{c} \begin{array}{cc} X\left(t;x,\alpha_{1}\left(u_{2}\right),u_{2}\right)\in M\left(t\right) & t\in \left[0,𝓣\left(x,\alpha_{1}\right)\right] \end{array} \end{array}.$$ (2)

In an actual pursuit game situation, the pursuer expects the expression to be true. The game can be defined in five different levels: capturable, locally capturable, globally capturable, small time locally capturable (STLC), and small time globally capturable (STGC). We define

$$\begin{array}{c} \{\begin{array}{l} P\left(t;M\right)=\left\{x\in R^{n}\text{|}\exists \alpha_{1}\in A_{1},T\left(x,\alpha_{1}\right)\leq t\right\},\\ P\left(M\right)=P\left(\infty ;M\right),\\ M\subseteq P\left(M\right). \end{array} \end{array}$$ (3)

Then the capturability can be described as follows (Yong, 1986):

$$\begin{array}{c} \{\begin{array}{l} locally\;capturable\;\Leftrightarrow \;\exists 𝒪\left(M\right)\subseteq P\left(\varepsilon ;M\right),\\ globally\;capturable\;\Leftrightarrow \;R^{n}\subseteq P\left(M\right),\\ STLC\;\Leftrightarrow \;\forall \varepsilon >0,\;\exists 𝒪\subseteq P\left(\varepsilon ,M\right),\\ STGC\;\Leftrightarrow \;\forall \varepsilon >0,\;R^{n}\subseteq P\left(\varepsilon ;M\right). \end{array} \end{array}$$ (4)

For any $x\in R^{n}$ , $a_{1}\in A_{1}$ , and $u_{2}\in U_{2}$ , the minimum terminating time T(x) can be defined as follows:

$$\begin{array}{c} \{\begin{array}{l} T\left(x;\alpha_{1}\left[u_{2}\right],u2\right)=\text{inf}\{t\geq 0\mathrm{|d}\left(X\left(t;x,\alpha_{1}\left[u_{2}\right],u_{2}\right),M\right)=0,\\ 𝓣\left(x\right)=\inf_{\alpha_{1}\in A_{1}}\;\sup_{u_{2}\in U_{2}}\;T\left(x;\alpha_{1}\left[u_{2}\right],u_{2}\right). \end{array} \end{array}$$ (5)

Then, we introduce the cost function $J\left(x;\alpha_{1}\left[u_{2}\right],u_{2}\right)$ :

$$\begin{array}{c} J\left(x;\alpha_{1}\left[u_{2}\right],u_{2}\right)=\overset{T\left(x;\alpha_{1}\left[u_{2}\right],u_{2}\right)}{\underset{0}{\int }}e^{-s}ds=1-e^{-T\left(x;\alpha_{1}\left[u_{2}\right],u_{2}\right)} \end{array}$$ (6)

The projective of the pursuer in the different pursuit games is to minimize the terminating time and the cost function when the game is capturable.

Symmetrically, the problem for the evader can be proposed in the following way:

$$\begin{array}{c} \{\begin{array}{ll} \dot{X}\left(t\right)=f\left(X\left(t\right),u_{1}\left(t\right),\alpha_{2}\left[u_{1}\right]\left(t\right)\right) & t\in \left[0,\infty \right),\\ X\left(0\right)=x & x\in R^{n}. \end{array} \end{array}$$ (7)

Considering M as the terminating set and $R^{n}\setminus M$ as the survival set. For any $x\in R^{n}\setminus M$ and any $u_{1}\in U_{1}$ , find an $\alpha_{2}\in A_{2}$ that makes the following expression true:

$$\begin{array}{c} \begin{array}{cc} X\left(t;x,u_{1},\alpha_{2}\left[u_{1}\right]\right)\notin M & t\in \left[0,\infty \right) \end{array} \end{array}$$ (8)

Correspondingly, the ability to evade (Lin, 1992) can be summarized as evadable and uniformly evadable. If a game is considered to be evadable from M , for any $u_{1}\in U_{1}$ and $x\in R^{n}\setminus M$ , there exists an $\alpha_{2}\in A_{2}$ . For those games to be uniformly evadable from M , there exists a δ>0 and an $\alpha_{2}\in A_{2}$ , the following expression holds:

$$\begin{array}{c} \begin{array}{cc} d\left(X\left(t;x,u_{1},\alpha_{2}\left[u_{1}\right]\right),M\right)\geq \delta , & \forall t\geq 0,\;u_{1}\in U_{1} \end{array} \end{array}.$$ (9)

In the evasion circumstances, the minimum terminating time and the cost function have similar definition to those in pursuit games. The purpose of the evader is to find out an $\alpha_{2}$ in the evadable game with less terminating time and cost.

$$\begin{array}{c} \begin{array}{l} T\left(x;u_{1},\alpha_{2}\left[u_{1}\right]\right)=\inf\left\{t>0X\left(t;x,u_{1},\alpha_{2}\left[u_{1}\right]\right)\in M\right\},\\ J\left(x;u_{1},\alpha_{2}\left[u_{1}\right]\right)=\overset{T\left(x;u_{1},\alpha_{2}\left[u_{1}\right]\right)}{\underset{0}{\int }}e^{-s}ds=1-e^{-T\left(x;u_{1},\alpha_{2}\left[u_{1}\right]\right)}. \end{array} \end{array}$$ (10)

The idea of the cost function is to minimize the cost of the evader, provided it can escape successfully. Later in this article, a reward mechanism shares the similar philosophy with the cost function here.

## 4 Modeling and Kinematic Analysis of Dog Sheep Game

### 4.1 Kinematic Analysis of Dog Pursuit Game

In the dog sheep game, the game environment is set to be a circle of radius R in which the dog runs at a constant speed of $V_{d}$ . The sheep also runs at a constant speed of $v_{s}$ , while its original position is set to be randomly distributed inside the circle. For convenience, given the dog and the sheep coordinates of $\left(R_{d},\theta_{d}\right)$ and $\left(r_{s},\theta_{s}\right)$ , $\left(R_{d}=R,r_{s}\in \left[0,R\right),\theta_{d},\theta_{s}\in \left[0,2\pi \right)\right)$ . In case if the sheep aborts its attempt to escape, the distance between the sheep and the center of the circle $r_{s}$ is constrained to be monotonically non-decreasing. It is assumed that the dog and the sheep both have the ability to adjust their heading directions without losing any speed. The game terminates as soon as the sheep gets caught or successfully escapes.

Under the aforementioned conditions, this section concentrates on the optimal pursuit strategy for the dog that maximizes its chance of successful pursuit and the kinematic evasion policy for the sheep, provided the dog adopts the optimal tactics. When the sheep escapes from the circle, the quickest way is to run outward along the radius where it is located. Hence, the ideal situation for the dog is to arrive at the intersection of the certain radius and the circle $\left(R,\theta_{s}\right)$ sooner than the sheep does, supposing that the sheep does not adjust its heading direction, which is not often the case. Therefore, the heading direction of the dog may change dynamically, but it is always the one that reduces the inferior angle Δθ formed by the radii where the dog and the sheep are located. Thus, the pursuit policy $\alpha_{1}$ for the dog can be summarized as follows:

$$\begin{array}{c} \{\begin{array}{l} \theta_{s}-\theta_{d}\in \left(-2\pi ,-\pi \right]\cup \left(0,\pi \right]\;\;\;The\;dog\;runs\;clockwise,\\ \theta_{s}-\theta_{d}\in \left(-\pi ,0\right)\cup \left(\pi ,2\pi \right)\;\;\;The\;dog\;runs\;anticlockwise,\\ otherwise\;\;\;Stay\;where\;it\;is. \end{array} \end{array}$$ (11)

**FIGURE 1:**
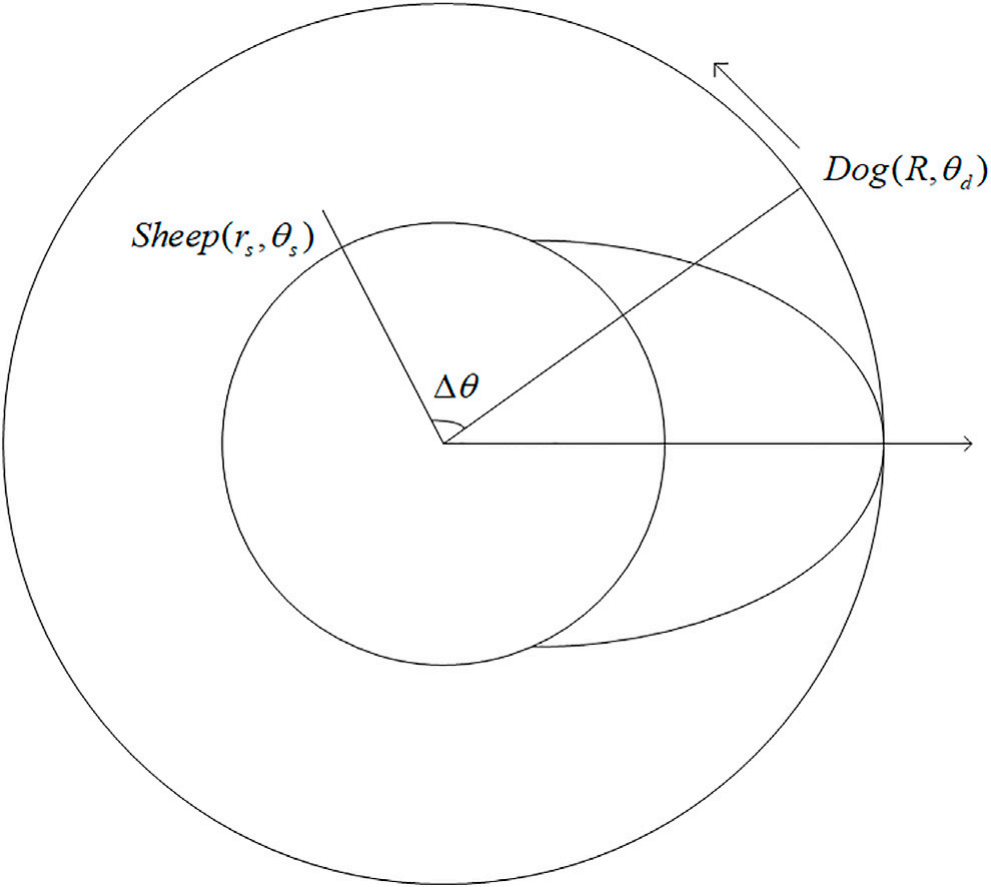
Diagram of the dog’s pursuit strategy.

From the perspective of differential games, the dog’s kinematic pursuit strategy $\alpha_{1}$ simply depends on the relative position of the sheep and the dog. Figure 1 portrays the pursuit strategy for the dog.

**FIGURE 2:**
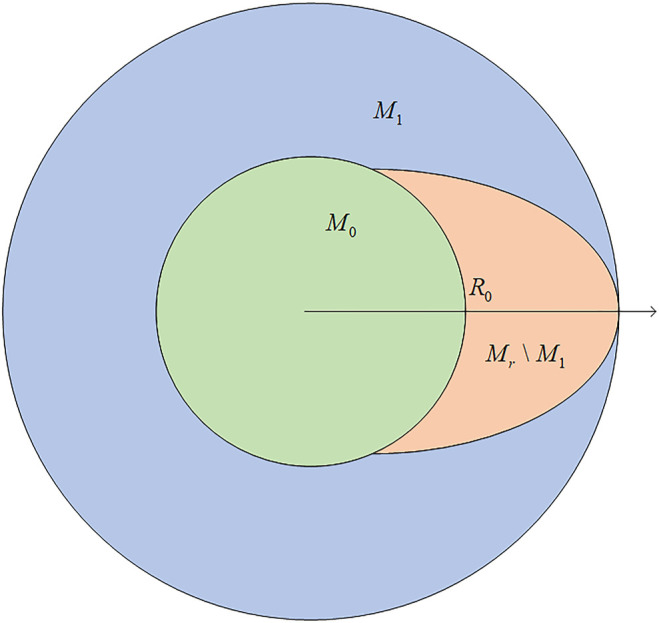
Sheep divided into three parts based on its relative position.

Considering the influence of the relative position of the sheep on the pursuit strategy, the capturability of the game needs to be discussed based on different origin positions of sheep. This inferior angle Δθ is determined by the angular velocity of the dog and the sheep, each being represented as $\omega_{d}\text{ and }\omega_{s}$ , respectively. The closer the sheep is to the circle, the slower will be its angular velocity $\omega_{s}$ . Correspondingly, $\omega_{s}$ may go to infinity as long as the sheep is close enough to the center of the circle $\left(r_{s}\to 0\right)$ . Thus, there exists an $r_{0}$ that meets the condition $\omega_{s}=\omega_{d}$ if $r_{s}=r_{0}$ . The set $M_{0}=\left\{\left(r_{s},\theta_{s}\right)|0\leq r_{s}<r_{0}\right\}$ , which is the green part in Figure 2, and the sheep located in it is capable to run at a larger angular velocity. This means the sheep has the ability to adjust Δθ as it wishes. Supposing the sheep has infinite physical power, it could run to the place with the greatest chance of escaping and then start running outward. So, when the sheep is born inside the circle of radius $r_{0}$ , the game is capturable when the sheep has no way of escaping if the following expression holds:

$$\begin{array}{c} \frac{R-r_{0}}{v_{s}}\geq \frac{\pi R}{V_{d}} \end{array}.$$ (12)

This means the sheep could not escape even at the place with the greatest chance of escaping $\left\{\left(r_{s},\theta_{s}\right)||\theta_{s}-\theta_{d}|=\pi \right\}$ .

Running inside the ring defined as $M_{r}$ formed by the outside circle and the circle of radius $r_{0}$ , the sheep’s angular velocity $\omega_{s}$ is smaller than $\omega_{d}$ . Considering the condition in which sheep is born inside this ring, there exists a set where any sheep located in it has no chance of escaping as long as the dog adopts the optimal pursuit strategy. We define this set as $M_{1}$ and it can be represented as follows:

$$\begin{array}{c} M_{1}=\left\{\left(r_{s},\theta_{s}\right)r_{0}\leq r_{s}<R,\;\left|\theta_{s}-\theta_{d}\right|\leq \frac{\left(R_{d}-r_{s}\right)V_{d}}{v_{s}R_{d}}\right\} \end{array}.$$ (13)

The game is capturable to the pursuer when sheep is located in $M_{1}$ , which is the red part in Figure 2. For the sheep located in $M_{r}\backslash M_{1}$ , which is the blue part in Figure 2, there exist some strategies that make it impossible for the dog to chase it down.

Figure 2 portrays the sheep in different parts of the circle to help better analyze the capturability of the game.

### 4.2 Kinematic Modeling of Sheep Evasion Game

In this part of the article, we focus on the kinematic evasion model of sheep in the dog sheep game under the circumstances that the dog adopts the optimal strategies to chase down the sheep. As discussed earlier, kinematic analyses for the sheep located in $M_{0},M_{1},\text{ and }M_{r}\backslash M_{1}$ should be separately conducted.

#### 4.2.1 Sheep Located in $M_{0}$

For the sheep whose initial states x are in $M_{0}$ , its kinematic evasion models can be uniformly discussed, since its relatively larger angular velocity endows it the ability to adjust Δθ and $r_{s}$ . This simplifies the analysis of game’s ability to evade and sheep’s evasion strategy from $M_{0}$ to those from $\left\{\left(r_{s},\theta_{s}\right)|r_{s}=r_{0}\right\}$ . As discussed before, the game is evadable if the following expression holds:

$$\begin{array}{c} \frac{R-r_{0}}{v_{s}}<\frac{\pi R}{V_{d}} \end{array}.$$ (14)

Assuming the game is evadable from $M_{0}$ , the strategy for sheep is to utilize its advantage to the dog in angular velocity to change the angle Δθ until the following evasion condition is satisfied:

$$\begin{array}{c} \begin{array}{cc} \frac{R-r_{s}}{v_{s}}<\frac{\left|\theta_{s}-\theta_{d}\right|R}{V_{d}} & r_{s}\in \left[0,r_{0}\right] \end{array} \end{array}.$$ (15)

Once the aforementioned expression holds, the evasion strategy for the sheep is to run straight outward.

#### 4.2.2 Sheep Located in $M_{1}$

Next, we focus on the sheep born inside $M_{1}$ . Theoretically speaking, it has no chance of escaping, provided the dog makes no mistake, that is, for any $\alpha_{2}\in A_{2}$ , $x\in M_{1}$ , there exists an $u_{1}\in U_{1}$ , and $M_{1}\cap \left(R_{n}\backslash \;M\right)=\emptyset \;$ holds. This means the game is not evadable from $M_{1}$ .

#### 4.2.3 Sheep Located in $M_{r}\backslash M_{1}$

**FIGURE 3:**
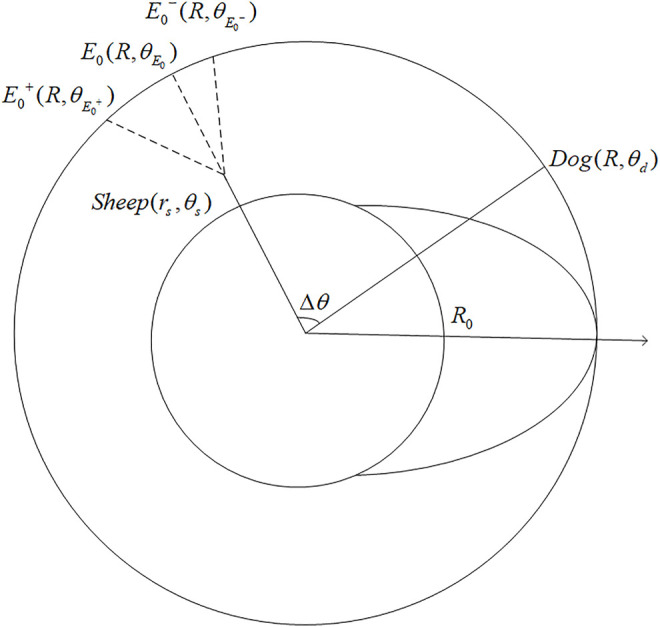
Diagram of evasion strategy for sheep located in $M_{r}\backslash M_{1}$ .

The only one scenario remains is when the sheep is born inside $M_{r}\backslash M_{1}$ . In this scenario, the sheep has the opportunity to escape successfully in many different ways, among which running outward along the radius where it is located is the quickest. In the meantime, the intersection of the circle and the certain radius $E_{0}\left(R,\theta_{E_{0}}\right)$ is defined. There exists an open neighborhood $\left(\theta_{E_{0}^{-}},\theta_{E_{0}^{+}}\right)$ of $\theta_{E_{0}}$ such that for the sheep running straight toward the point inside this neighborhood, the dog would not be able to hunt it down. Define $E_{0}^{-}\left(R,\theta_{E_{0}^{-}}\right)$ , $E_{0}^{+}\left(R,\theta_{E_{0}^{+}}\right)$ , and the distance between the sheep and $E_{0}^{-}$ , $E_{0}^{+}$ as $d_{sE_{0}^{-}}$ , $d_{sE_{0}^{+}}$ for convenience. Specific details are shown in Figure 3.

We are able to solve $d_{sE^{-}}\text{ and }d_{sE^{+}}$ first and then $\theta_{E_{0^{-}}}\text{ and }\theta_{E_{0^{+}}}$ :

$$\begin{array}{c} \{\begin{array}{l} d_{sE_{0}^{-}}=\sqrt{r_{s}^{2}\sin^{2}\left(\theta_{s}-\theta_{E_{0}^{-}}\right)+{\left(R-r_{s}\cos\left(\theta_{s}-\theta_{E_{0}^{-}}\right)\right)}^{2}}\\ d_{sE_{0}^{+}}=\sqrt{r_{s}^{2}\sin^{2}\left(\theta_{s}-\theta_{E_{0}^{+}}\right)+{\left(R-r_{s}\cos\left(\theta_{s}-\theta_{E_{0}^{+}}\right)\right)}^{2}} \end{array} \end{array}.$$ (16)

By definition, if the sheep runs straight toward $E_{0}^{-}$ and $E_{0}^{+}$ , the dog would catch it just in time. Therefore, we have

$$\begin{array}{c} \{\begin{array}{l} \frac{d_{sE_{0}^{-}}}{v_{s}}=\frac{\left|\theta_{d}-\theta_{E_{0}^{-}}\right|R}{V_{d}}\\ \frac{d_{sE_{0}^{+}}}{v_{s}}=\frac{\left|\theta_{d}-\theta_{E_{0}^{+}}\right|R}{V_{d}} \end{array}\Rightarrow \{\begin{array}{l} d_{sE_{0}^{-}}=\frac{\left|\theta_{d}-\theta_{E_{0}^{-}}\right|v_{s}R}{V_{d}}\\ d_{sE_{0}^{+}}=\frac{\left|\theta_{d}-\theta_{E_{0}^{+}}\right|v_{s}R}{V_{d}} \end{array} \end{array}.$$ (17)

So, the polar coordinates of $E_{0}^{-}$ and $E_{0}^{+}$ can be solved as follows:

$$\begin{array}{c} \{\begin{array}{l} \theta_{E_{0}^{-}}=\theta_{d}-sgn\left(\sin\left(\theta_{s}-\theta_{d}\right)\right)\cdot \frac{V_{d}\sqrt{r_{s}^{2}\sin^{2}\left(\theta_{s}-\theta_{E_{0}^{-}}\right)+{\left(R-r_{s}\cos\left(\theta_{s}-\theta_{E_{0}^{-}}\right)\right)}^{2}}}{v_{s}R}\\ \theta_{E_{0}^{+}}=\theta_{d}+sgn\left(\sin\left(\theta_{s}-\theta_{d}\right)\right)\cdot \frac{V_{d}\sqrt{r_{s}^{2}\sin^{2}\left(\theta_{s}-\theta_{E_{0}^{+}}\right)+{\left(R-r_{s}\cos\left(\theta_{s}-\theta_{E_{0}^{+}}\right)\right)}^{2}}}{v_{s}R} \end{array} \end{array}.$$ (18)

For the sheep whose initial position is $M_{r}\backslash M_{1}$ , if it does not run straight to the inferior arc formed by $E_{0}^{-}$ and $E_{0}^{+}$ , the evasion time is limited to the open interval $\left(\frac{R-r_{s}}{v_{s}},min\left(\frac{d_{sE_{0}^{-}}}{v_{s}},\frac{d_{sE_{0}^{+}}}{v_{s}}\right)\right)$ or it would miss the chance of escaping.

## 5 Implementation of Deep Reinforcement Learning Methods for Dog Sheep Game

### 5.1 Technical Foundation of Deep Reinforcement Learning Methods

Deep reinforcement learning (DRL) has shown its advantages in various games over the past few years, especially since DeepMind creatively introduced DQN (2013) (Jiang et al., 2021a), which combines reinforcement learning and deep learning. Recently, many similar research studies on implementations of DRL for different game scenarios emerged.

DQN was initially presented by DeepMind (2013) (Jiang et al., 2021a) to play the Atari video game, and it successfully learned the control policy and remarkably outperformed human-level performance. Excellent as DQN may seem, adjustments are still required when it is applied to other game scenarios such as the design of reward systems and the adoption of replay buffers (2018) (Wei et al., 2018).

As a value-based method, it evaluates each possible action at certain stages in the game based on the Q-value. When an agent is at state $s_{t}$ and takes an action $a_{t}$ , $Q\left(s_{t},a_{t}\right)$ is used to estimate the value at this certain time step t . Then it chooses the suitable action based on the action select strategy. After the execution of action a at state s , the reward is $r_{t}$ , and the game goes to the next state $s_{t+1}$ . In order to enable the neural network to learn from experiences, the experience replay is introduced. A four-tuple $\left(s_{t},a_{t},r_{t},s_{t+1}\right)$ is stored in the experience replay buffer, and the network would sample and learn from it.

### 5.2 Implementation of Deep Q Network for Dog Sheep Game

In this subsection, we propose DQN to the dog sheep game. Technical details and model optimizations will be elaborated as well.

#### 5.2.1 Environment Settings

Environment settings are of great significance since the environment is the one the agent interacts with. To simulate the dog sheep game, our game environment consists of basic parameters, initializations, and environment updates.

##### 5.2.1.1 Basic Parameters and Initializations

In the dog sheep game environment, the radius of the circle R is set to be 200. Since determining whether the game terminates requires judging whether they have physical contact or not, it would be inappropriate to consider both sides as particles. Apart from the sizes of both agents, their running speed should be specified as well. For relatively bigger sizes or a smaller dog sheep speed ratio, the difficulty for sheep to evade increases considerably. Hence, proper sizes and speed should be initialized to prevent the environment from being partial to one side in the pursuit–evasion game. After a number of experiments and simulations, we set the size of both agents as 10 and the dog speed $V_{d}=16$ to make sure each episode ends at around 11 steps. The speed of the sheep is determined by the speed ratio $\eta =\frac{V_{d}}{v_{s}}$ , which is a key parameter to evaluate the environment difficulty. The exact value of η will be given later.

##### 5.2.1.2 Environment Updates

As the simulation proceeds, whether the game is terminated should be checked every time the game enters a new time step. Then the sheep and the dog could take actions resulting in the changes of their positions if the game is not finished. Their next positions can be deduced using their current positions and their heading directions.

As it is discussed previously, the dog’s heading direction is decided by its capture strategy, while the sheep’s heading direction is something to be learned in the DRL model. For DQN, the action space is discrete. If we allow the sheep to adjust its deflection angle in degree, this means there would be 181 different possible actions at each time step. This will lead to the exponential growth of the requirement for computational power. Therefore, we adjust the action space and set it to be two dimensional and come up with an attenuation mechanism of the deflection angle. The sheep in the game is only left with the choice to turn left or right, while the steering angle $\theta_{sa}\in \left[-\frac{\pi }{2},\frac{\pi }{2}\right]$ is determined by a function set in advance. Given the fact that $\omega_{s}$ decreases as $r_{s}$ grows and equals to $\omega_{d}$ when $r_{s}=r_{0}$ , it may be quite costly if the sheep chose to make a 90° turn when it is close to the circle of the radius R . For the sheep inside or on the circle of radius $r_{0}$ , the value of $\theta_{sa}$ has no negative influence on their chances of escaping. However, due to the fact that $\omega_{s}$ is at a disadvantage when $r_{s}\in \left(r_{0},R\right)$ , the sheep should make its escape as soon as possible, which means $\theta_{sa}$ should be relatively small. As discussed previously, we introduce the steering angle function as follows:

$$\begin{array}{c} \theta_{sa}=\frac{\pi }{2}\cdot \underset{Distance\;discount}{\left(1-\frac{r_{s}}{R}\right)}\cdot \underset{Angle\;discount}{\left|\cos\left(0.5\left(\theta_{s}-\theta_{d}\right)\right)\right|} \end{array}.$$ (19)

The attenuation mechanism of the deflection angle consists of a distance discount and an angle discount. As the sheep runs outward, its maximum steering angle is restricted by the distance discount. This prevents the sheep from making sharp turns at unsuitable locations. However, it would be unnecessary for the sheep that already is an ideal initial position to turn its heading direction. So the angle discount is introduced to prevent that from happening. When the sheep is at an ideal position where $\left|\theta_{s}-\theta_{d}\right|$ like π , the angle discount is 0. This means the sheep does not have to change its heading direction at all. The angle discount is 1 when $\left|\theta_{s}-\theta_{d}\right|$ is 0 or 2π , resulting in a relatively large steering angle.

#### 5.2.2 Reward Mechanism

Agents in reinforcement learning models interact with environments, striving for high rewards. Therefore, a carefully designed reward mechanism is required when DQN is applied to a new scenario. A poorly designed one may lead to ineffectiveness and incapacity to learn the optimal strategy. The dog sheep game is also a zero-sum game, thus escaping successfully is of utmost significance to the sheep. Whenever the sheep escapes successfully, the terminate reward of 10 is given. On the contrary, once the sheep gets caught, the reward would be −10. This basic reward mechanism encourages the sheep to find a way to escape from the circle without being captured and win the pursuit–evasion game.

In the evasion game theory, the cost function is $J\left(x;u_{1},\alpha_{2}\left[u_{1}\right]\right)=\overset{T\left(x;u_{1},\alpha_{2}\left[u_{1}\right]\right)}{\underset{0}{\int }}e^{-s}ds$ . As time passes by, the cost increases correspondingly. Apart from the terminate reward, another reward should be given in each step to help minimize the evasion cost. Therefore, we have come up with a time-out reward strategy. If the sheep stays in the circle for too long, it will be given a negative reward. This reward should take the different origin locations into consideration. The sheep that is close to the circle needs less time for evasion than the sheep located around the center of the circle. So the time-out reward strategy is designed based on the origin location and the current time step.

$$\begin{array}{c} R_{to}=\{\begin{array}{ll} \rho log_{\frac{v_{s}}{V_{d}}}\left(t-\frac{R-r_{s}}{v_{s}}\right) & t-\frac{R-r_{s}}{v_{s}}>1,\\ 0 & else. \end{array} \end{array}$$ (20)

When the time is still within $\frac{R-r_{s}}{v_{s}}$ , which is the minimum evasion time for the sheep, there will be no reward given. Once the time exceeds the minimum evasion time, the negative reward is given and will increase as time passes by. Adding up this negative reward effectively prevents the sheep from lingering in the circle.

#### 5.2.3 Model Architecture

**FIGURE 4:**
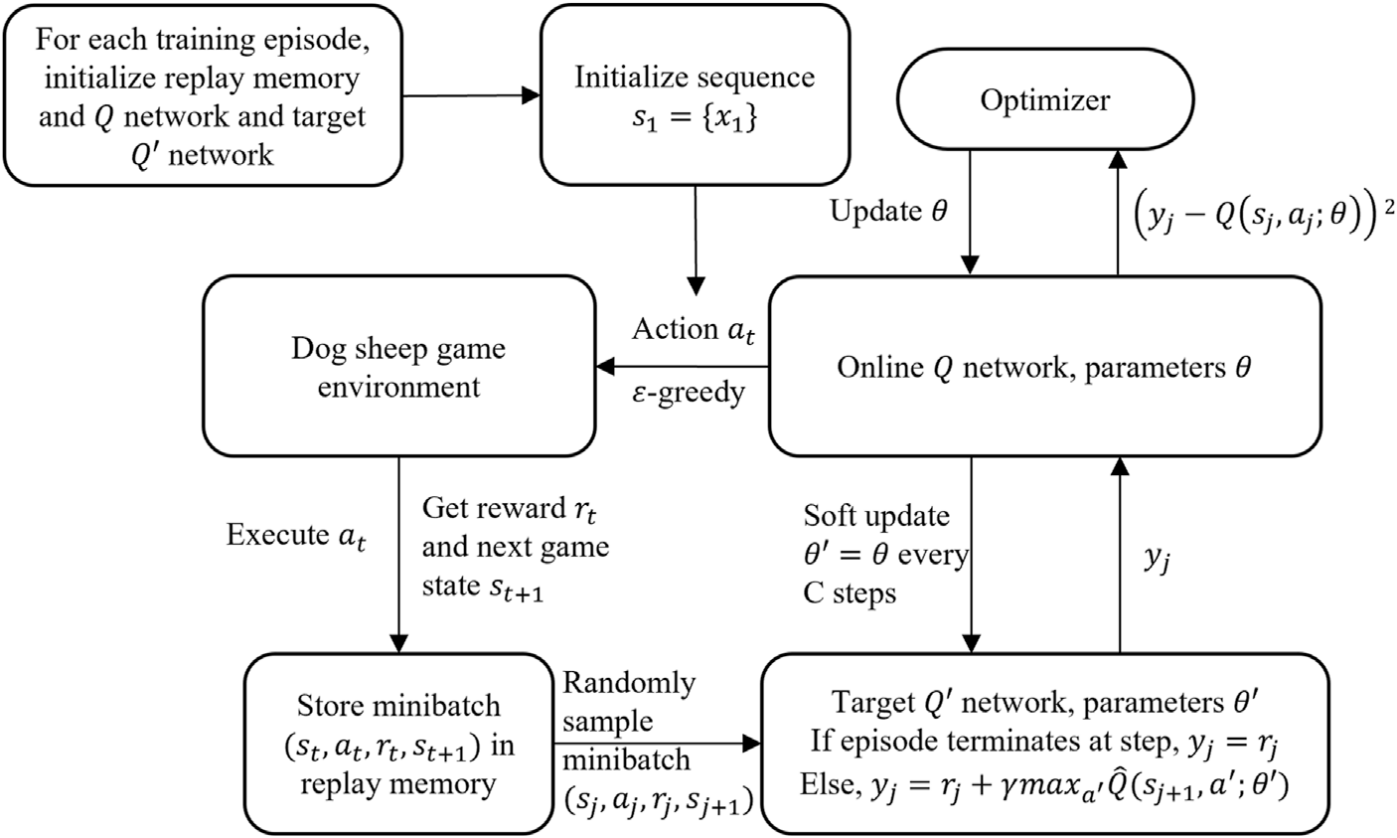
Flowchart of DQN training.

DQN has shown its capability to train the agent to play video games. It trains the agent to learn through the Q value throughout the training episode. Its structure is displayed in Figure 4.

To observe the game state, an MLP works as the state encoder. At each time step, based on the current game state $s_{t}$ observed, the neural network, whose parameters are randomly initialized, is able to calculate the Q value of every possible action and therefore come out of the best action with the highest potential rewards. Among all the actions, the agent chooses one of them based on the ε -greedy strategy. This means the agent would go for the action with the highest Q value at the possibility of 1−ε , while one of the remaining actions is chosen randomly and is executed at the possibility of ε . Introducing the ε -greedy strategy helps the agent learn from those actions that seem less attractive but may lead to higher rewards in the long run. This is of immense help at the threshold of each training episode. The pseudo code of DQN is shown in Algorithm 1.

Algorithm 1 Deep Q-learning Network.

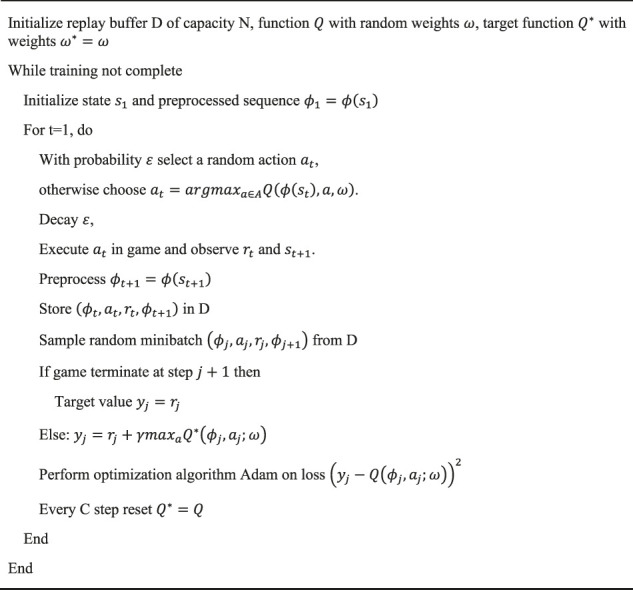

Once the action $a_{t}$ is selected, the reward $r_{t}$ is given and the next game state $s_{t+1}$ is observed. A four-tuple $\left(s_{t},a_{t},r_{t},s_{t+1}\right)$ is stored in the experience replay buffer. The optimizer takes a random sample from the replay buffer and utilizes it to update the parameters of the online Q network. This endows the agent the ability to learn from the valuable experience and helps optimize the learning process. Every several steps, the parameters of target Q network get a soft update from the online Q network.

### 5.3 Implementation of Deep Deterministic Policy Gradient for Dog Sheep Game

Compared with DQN, the action space of agents in DDPG is continuous. As discussed before, although the steering angle is carefully considered and designed with a distance discount and an angle discount, the action space is discrete after all. The discrete action space with only 2 choices is just a compromise to insufficient computational power. It is always better to have a continuous action space.

**FIGURE 5:**
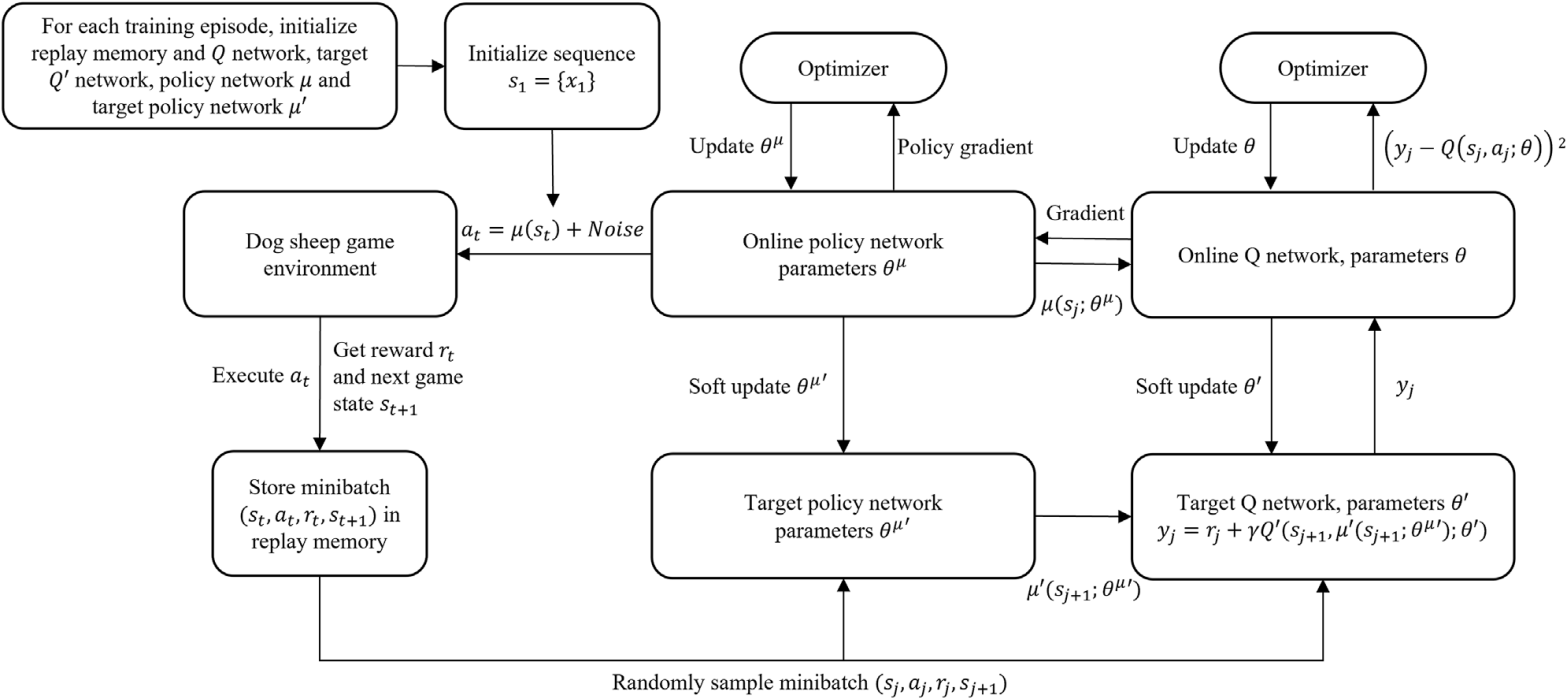
Flowchart of DDPG training.

DDPG has an actor-critic architecture, where the actor network optimizes its policy and the critic network estimates the Q-value for current policy. The training process is displayed in Figure 5. The online Q network estimates the Q-value based on the sampled four-tuple $\left(s_{t},a_{t},r_{t},s_{t+1}\right)$ . Like DQN, the experience replay and the reward mechanism work similarly in DDPG.

While the Q network updates its parameters, the online policy network, which is an MLP, updates its parameters at the same time. Every couple of steps, the parameters of both target Q and policy networks each gets a soft update from the online Q and policy networks. The details of DDPG are summarized in the Algorithm 2.

Algorithm 2 Deep Deterministic Policy Gradient (DDPG).

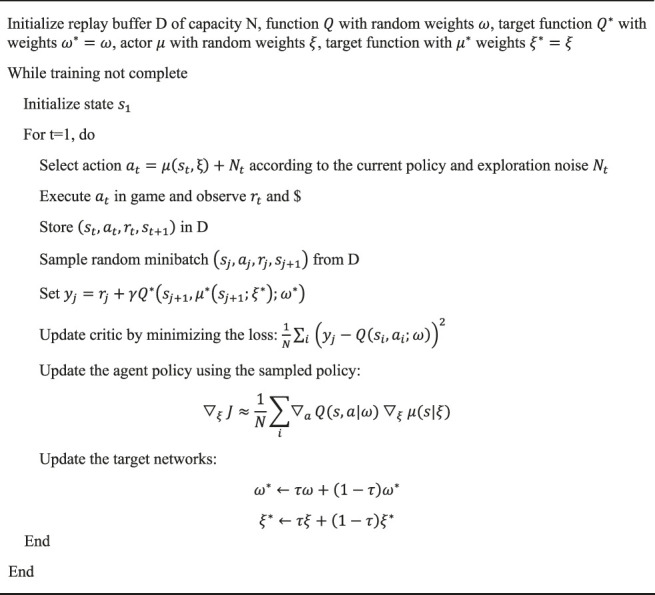

### 5.4 Learning Ability Evaluation

The implementation of the aforementioned methods is done when the speed ratio is 2 and the size of both agents is 10. This section focuses on the learning abilities of DRL methods under different environment difficulties. Among all the parameters, we choose the escape probability of baseline model and the escape rate improvement to quantify the environment difficulty and the learning ability. In this scenario, the size is 10 and the dog speed $V_{d}$ is 16. Sheep speed $v_{s}$ fluctuates according to the speed ratio η . If the game is evadable, the speed ratio η has

$$\begin{array}{c} \frac{\pi R}{V_{d}}>\frac{R-r_{0}}{v_{s}} \end{array},$$ (21)

$$\begin{array}{c} \frac{R}{V_{d}}=\frac{r_{0}}{v_{s}} \end{array},$$ (22)

$$\begin{array}{c} \eta =\frac{V_{d}}{v_{s}}<\pi +1 \end{array}.$$ (23)

This article only focuses on the performance of DRL methods while speed ratio is η∈(1,π+1) . Each speed ratio η corresponds to an escape probability. A baseline model is introduced to help quantify the environment difficulty. The sheep in this baseline model has no evasion policy and runs straight outward regardless of the condition. After implementing the DRL methods under various environment difficulties, the escape rate improvement is utilized to evaluate their learning ability.

## 6 Deep Reinforcement Learning Training Results

When implementing the DQN model, an MLP of three layers with game states as input is adopted to evaluate the Q value of each action. ε in the ε -greedy strategy decays linearly from 0.9 to 0.01, which allows the agent to attempt various actions when it is relatively ignorant and gradually prefers the action with the highest potential reward. The experiencing replay buffer is set to be 10,000. The discount factor in Algorithm 1 is set to be 0.95.

In our experiment, we introduce several baseline models whose key characteristics are displayed in the following Table 1. The DQN-T, DDPG-T, and DQN-A model are tested to evaluate the effectiveness of the time-out reward strategy and the attenuation mechanism.

**TABLE 1:** Models adopted in our experiments.

Type	Model	Characteristic
Models with discrete action space	DQN-T	DQN model without the time-out reward strategy
	DQN-A	DQN model without the attenuation mechanism of the deflection angle
	Refined DQN	Refined deep Q network model
	Baseline 1	Sheep run straight outwards
Models with continuous action space	DDPG	Deep deterministic policy gradient
	DDPG-T	DDPG model without the time-out reward strategy
	Baseline 2	Sheep take random deflection angle

**FIGURE 6:**
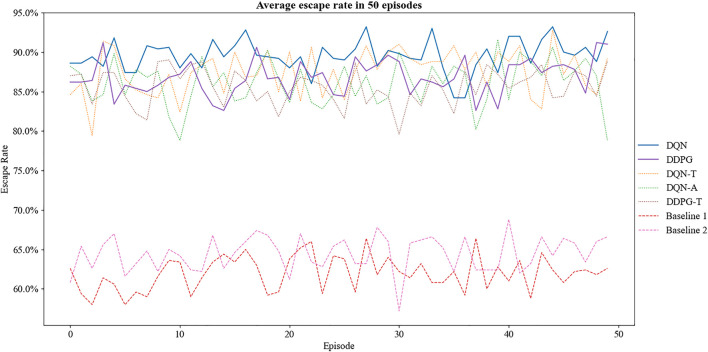
Average escape rate in 50 episodes.

For the DQN models and the DDPG models, the number of training episodes is set to be 1,500 and that of simulation episodes is 500. The dog speed is 16, and the sheep speed is 8. Experiments show that the DDPG and DQN models both outperform the baseline models. Compared to the two baseline models, all DRL methods improve the escape rate by a large margin of about 50%. The escape rate of each model in 50 rounds of simulation with various random speed is plotted in Figure 6.

**FIGURE 7:**
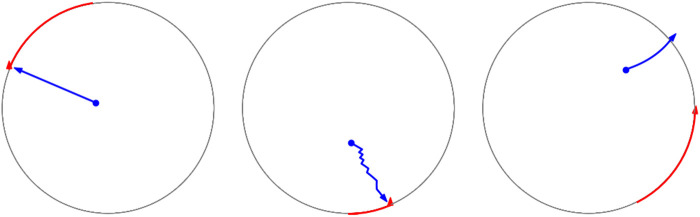
Trajectories of sheep. **(A)** From left to right, each represents the trajectory of sheep from the baseline models 1 and 2 and the DRL model. **(B)** Blue curves are the trajectories of sheep, while the red ones are those of dogs.

It can be seen that the sheep trained by the refined DQN model has a greater chance of escaping than the DQN-T and DQN-A models. The DDPG model also achieves a higher escape rate than the DDPG-T model. This indicates the effectiveness of the time-out reward strategy. It is illustrated that the modifications and adjustments effectively increase the success rate of evasion and the performance of the DQN model. To better illustrate the differences in the DRL methods and the baseline models, the trajectories of sheep during their evasion process are shown in Figure 7. From left to right, each represents the trajectory of sheep from the baseline models 1 and 2 and the DRL model.

In addition, the performance difference between the refined DQN model and the DDPG model is quite narrow. Yet generally speaking, the refined DQN model outperforms the DDPG model by a small margin.

To better evaluate the performance of the aforementioned models, the average reward in 50 rounds of simulation is displayed in Table 2. Unsurprisingly, the average reward of each model corresponds to the average escape rate. Compared to the time-out reward strategy, the attenuation mechanism of the deflection angle causes greater enhancement for the DQN and DDPG models. This attenuation mechanism helps the DQN model achieve even better performance than the DDPG model.

**TABLE 2:** Average reward of different models.

Model	Average reward
Refined DQN	7.3601
DDPG	7.2507
DQN-T	6.6804
DQN-A	5.7600
DDPG-T	6.8300
Baseline 1	1.8237
Baseline 2	2.9427

**FIGURE 8:**
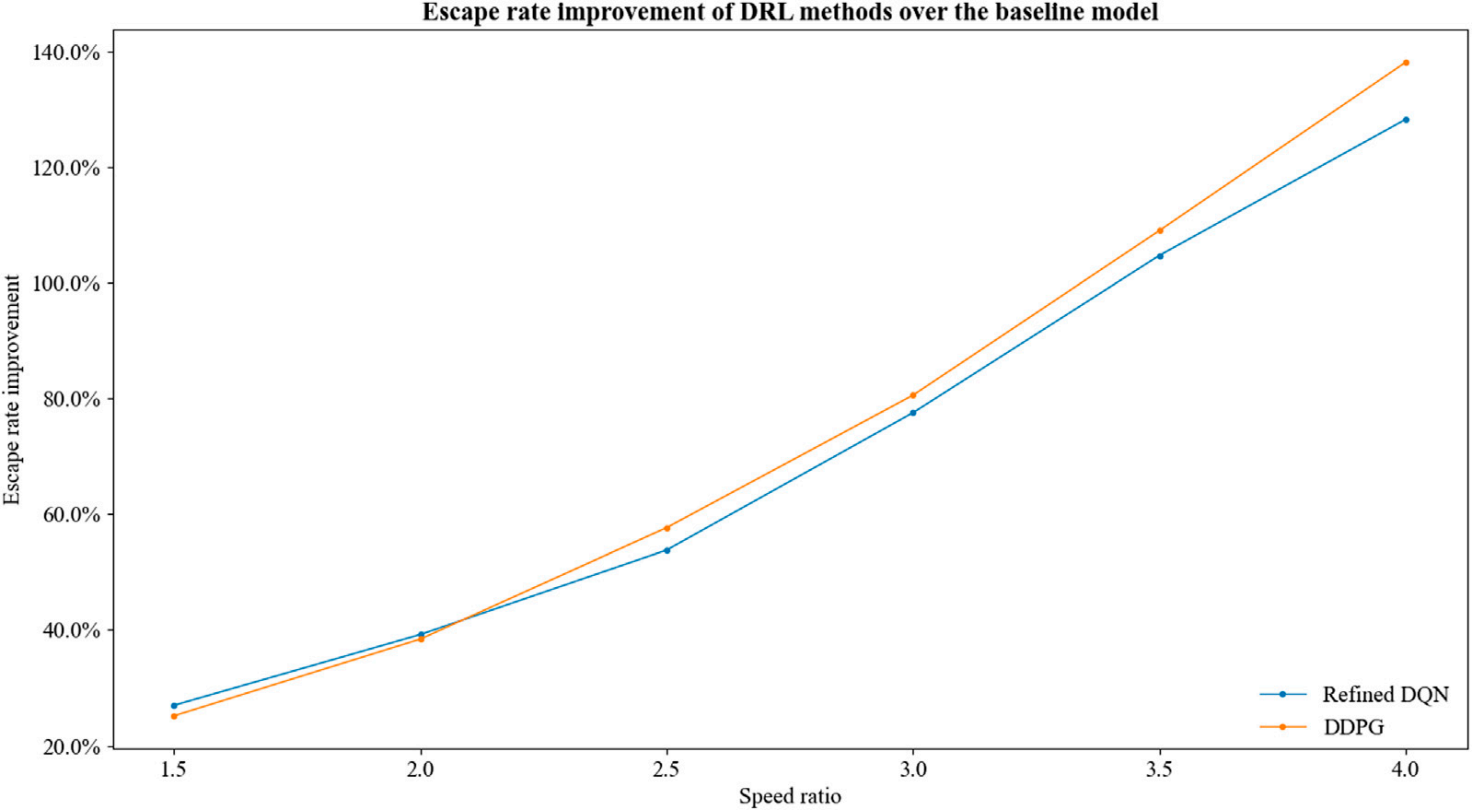
Escape rate improvement of DRL methods over the baseline model.

Furthermore, we manage to evaluate the learning abilities of the aforementioned DRL methods. For speed ratio η taken at the interval of 0.5 from 1.5 to 4, the average improvement of escape probabilities in 50 rounds of simulation is shown in Figure 8.

As the speed ratio increases, the game environment difficulty increases. Correspondingly, the average improvement of escape probability increases. The DRL models have stronger learning ability in harsh game environments.

## 7 Discussion

In this article, we first introduce a bio-inspired differential pursuit–evasion game: the dog sheep game. Our research explores the implementation of DRL methods for training the ignorant sheep to escape. Compared to other methods applied to differential pursuit–evasion games, DRL methods adopted are model-free and require less optimization.

Based on the traditional differential pursuit–evasion game theory, we come up with the kinematic model for the dog and the sheep. The dog’s strategy can be summarized as reducing the inferior angle formed by the radii where the dog and the sheep are located. The sheep’s strategy, which depends on its original location, is a bit more complicated. For a sheep that is theoretically evadable, it should run straight outward once the critical evasion conditions are satisfied.

Subsequently, we manage to adopt deep reinforcement learning methods to learn evasion strategy. In terms of the game environment settings, an attenuation mechanism of the deflection angle is applied due to the discrete action space of the DQN model. According to the idea of the cost function in differential evasion games, a time-out strategy is added to the reward mechanism. The aforementioned modifications show great improvement compared to the original DQN model and the baseline model. We also adopt the DDPG model, which allows the action space to be continuous. It has excellent performance as well. The refined DQN model outperforms the DDPG model by a small margin. The learning abilities of the DRL methods under different environment difficulties are assessed based on the improvement of the escape probabilities to the baseline model. Simulations indicate that they both have excellent learning ability in harsh environments.

This research shows that DRL methods are of great significance to differential pursuit–evasion games. Implementing these methods requires no manually designed features and less optimization for different game scenarios. The limitation of this research is the dog sheep game itself. This particular differential pursuit–evasion game scenario is simple. The DRL methods adopted by us are able to cope with more complex game scenarios, and we are looking forward to applying them to other games in the future.

## Data Availability

The original contributions presented in the study are available at https://github.com/LEOXC1571/DSG. Supplementary Material, further inquiries can be directed to the corresponding author.
